# Association between two single nucleotide polymorphisms of the Prostaglandin-Endoperoxide Synthase 1 and 2 genes and cell proliferative prostatic diseases in Lebanon

**DOI:** 10.18632/oncotarget.28710

**Published:** 2025-04-04

**Authors:** Brock J. Sheehan, Bryson Edwards, Ivanna Soto Medrano, Mohammed A. El-Saidi, Wissam R. Zaidan, Asmahan A. El-Ezzi, Ruhul H. Kuddus

**Affiliations:** ^1^Department of Biology, Utah Valley University, Orem, UT 84058, USA; ^2^Department of Strategic Management and Operations, Utah Valley University, Orem, UT 84058, USA; ^3^Radioimmunoassay Laboratory, Lebanese Atomic Energy Commission, Beirut, Lebanon; ^4^Department of Chemistry and Biochemistry, Lebanese University, Hadath, Lebanon

**Keywords:** PTGS1 and PTGS2 genes, COX-1 and COX-2 isozymes, single nucleotide polymorphism association study, prostate cancer, benign prostate hyperplasia (BPH)

## Abstract

The polymorphic genes PTGS1 and PTGS2 encode cyclooxygenases COX-1 and COX-2, respectively. Overexpression of these cyclooxygenases is linked to inflammation and neoplasms. This study investigated the potential association between the single nucleotide polymorphism (SNP) -842A>G (rs10306114) of the PTGS1 gene and SNP-765G>C (rs20417) of the PTGS2 gene with prostate cancer (PCa) and benign prostate hyperplasia (BPH). Blood leucocyte DNA from 56 healthy individuals, 61 individuals with PCa, and 51 individuals with BPH were genotyped using the PCR-RFLP method. Associations were inferred by calculating odds ratios (OR) and relative risks (RR) of genotype distributions and allele frequencies. The genotypes for both SNPs were in Hardy-Weinberg equilibrium for all groups. No significant association was observed between the A or G alleles or the AA, AG, or GG genotypes of the SNP-842A>G of the PTGS1 gene and prostatic diseases. However, the C allele of SNP-765G>C of the PTGS2 gene was significantly associated with an increased risk of BPH (OR = 2.30, *p*-value = 0.01). Differences in the ratios of GG/GC and GG/(GC+CC) genotypes also suggested a potential association between the C allele and PCa (*p*-value <0.1), and the combined affected (PCa+BPH) group (*p*-value <0.04). The small sample size and sampling from one ethnic group are limitations of this study.

## INTRODUCTION

Cyclooxygenases (COXs) 1 and 2 or Prostaglandin-Endoperoxide Synthases (PTGS) 1 and 2 (EC 1.14.99) are fatty acid oxygenase isozymes within the myeloperoxidase superfamily of enzymes. The PTGS1 and 2 genes evolved through gene duplication, and both genes are present in the genome of cnidarians (coral) and all chordates [1]. The PTGS1 gene is on chromosome 9 (9q33.2; 122,370,530-125,157,982) and encodes the isoenzyme COX-1 [2]. The PTGS2 gene is on chromosome 1 (1q31.1; 186,671,791-186,680,922) and encodes the isoenzyme COX-2 [2]. The two isozymes are about 60–65 % identical in their amino acid sequences [1]. Both genes are expressed in the prostate gland [3].

COX-1 typically functions in platelet aggregation, gastrointestinal mucosa protection, and vascular homeostasis, while COX-2 involves induction of inflammation and mitogenesis [4]. In general, COX-1 is considered constitutive, and COX-2 inducible isoform [1]. However, recent findings suggest that both isoforms can be constitutively expressed in certain tissues and may be induced under specific conditions [5, 6]. Gene knock-out experiments in mice have demonstrated that expression of the PTGS2 gene is essential, while the same for the PTGS1 gene plays complementary roles in embryonic, fetal, and neonatal survival [6]. Overexpression of both genes is implicated in cancers of various organs. For instance, COX-1 overexpression has been observed in cancers of the female breast [7], ovary [8], colon [9], and several other organs reviewed in [10]. Similarly, COX-2 overexpression has been noted in cancers of female breasts [7], colorectal tissues [11], prostate [12], and other organs reviewed in [13].

Both the PTGS1 and PTGS2 genes have several reported single nucleotide polymorphisms (SNPs) [14]. SNPs are common germline point mutations with a frequency >1% of the population and are generally presumed benign [15]. However, some SNPs can influence overall gene expression by altering protein structure and function through missense mutations, which may involve conservative or nonconservative amino acid changes. These SNPs can also affect transcription, translation, RNA and polypeptide grooming, and the stability of fully groomed mRNAs and proteins [16]. Certain SNPs in PTGS1 and PTGS2 genes have been linked to cancer [17, 18]. Our study focused on the SNP -842 A>G (i.e., rs10306114) of the PTGS1 gene and the SNP -765 G>C (i.e., rs20417) of the PTGS2 gene. These SNPs are located in the upstream proximal promoters of the genes, where transcription regulators may bind and influence the transcription rate. Additionally, covalent modifications of DNA and histones localized in this region may also affect the rate of transcription of the genes. Given that both genes are expressed in the prostate gland, we hypothesized that these SNPs could be associated with the risk of cell proliferative prostatic diseases.

Prostatic diseases characterized by cell proliferation include prostate cancer (PCa) and benign prostate hyperplasia (BPH). In PCa, transformed (neoplastic) epithelial cells proliferate, whereas in BPH, untransformed epithelial and stromal cells proliferate [19]. Globally, PCa is the second most frequently diagnosed cancer (after lung/bronchial cancer) and the third leading cause of cancer deaths (following lung/bronchial and liver cancers) among men [20]. The incidence and mortality rates of PCa have been rising in most countries [21]. BPH affects over 70% of males older than 60 [22], and its global prevalence has nearly doubled, from approximately 51.1 million in 2000 to about 95 million in 2019 [23].

Early diagnosis is crucial for successful therapeutic intervention in cancer, but PCa can be an insidious and difficult-to-diagnose disease. In 2019, about 30% of PCa diagnosed in the USA were in stage 3 or beyond [24]. Conversely, BPH presents disease signs early in its progression. Therefore, identifying genetic markers common to both PCa and BPH can be diagnostically valuable. This study indicates a significant association between the SNP -765 G>C of the PTGS2 gene and BPH and possibly PCa.

## RESULTS

### Distribution of genotype and allele frequency

The distribution of XX, Xx and xx genotypes for the SNPs -842 A>G of the PTGS1 gene and -765 G>C of the PTGS2 gene is presented in Table 1. All samples conform to Hardy-Weinberg equilibrium.

**Table 1:** The difference between the observed and expected ratio of the different genotypes for the SNP -842 A>G of the PTGS1 gene and the SNP -765 G>C of the PTGS2 gene in the control and affected groups

Sample	Observed			Expected			χ^2^
	XX	Xx	xx	XX	Xx	xx	
SNP -842 A>G of the PTGS1 gene							
Control	53	3	0	53.04	2.92	0.04	0.04
PCa	59	2	0	59.01	1.97	0.02	0.02
BPH	50	1	0	50.00	0.99	<0.01	<0.01
PCa + BPH	109	3	0	109.02	2.96	0.02	0.02
SNP -765 G>C of the PTGS2 gene							
Control	7	23	26	6.11	24.78	25.11	0.29
PCa	2	31	28	5.02	24.96	31.02	3.57
BPH	1	16	34	1.59	14.82	34.59	0.32
PCa +BPH	3	47	62	6.27	40.46	65.27	2.93

The degree of freedom for a balletic trait is 1, and the upper-tail critical value of χ^2^ distribution is 3.841.

The allele frequencies for the dominant (B) and recessive (b) alleles of the SNPs -842 A>G of the PTGS1 gene are detailed in Table 2. The recessive (b) allele is rare in the Lebanese population, with no bb genotype observed in the sample (Table 2). The allele frequency difference between the control and PCa groups is statistically insignificant (OR = 0.61, 95% CI is 0.10–3.69, *p*-value = 0.59). Similarly, the difference between the control and BPH groups is statistically insignificant (OR = 0.36, 95% CI is 0.04–3.51, *p*-value = 0.38). Furthermore, the difference between the control and the combined affected group (PCa+ BPH) is also statistically insignificant (OR = 0.49, 95% CI is 0.09–2.48, *p*-value = 0.39).

**Table 2:** The difference in the frequency of the alleles for the SNP -842 A>G of the PTGS1 gene and the SNP -765 G>C of the PTGS2 gene in the control and affected groups

SNP -842 A>G of the PTGS1 gene: Controls vs. BPH					
Alles	Control	Affected	OR	95% CI	*p*-value
Control vs. PCa					
b	3 (0.03)	2 (0.02)	0.61	0.10–3.69	0.59
B	109 (0.97)	120 (0.98)			
Control vs. BPH					
b	3 (0.03)	1 (0.01)	0.36	0.04–3.51	0.38
B	109 (0.97)	101 (0.99)			
Control vs. PCa +BPH)					
b	3 (0.03)	3 (0.01)	0.49	0.09–2.48	0.39
B	109 (0.97)	221 (0.99)			
SNP -765 G>C of the PTGS2 gene					
Control vs. PCa					
a	75 (0.67)	87 (0.71)	1.22	0.70–2.14	0.47
A	37 (0.33)	35 (0.29)			
Control vs. BPH					
a	75 (0.67)	84 (0.82)	2.30	1.21–4.38	0.01^*^
A	37 (0.33	18 (0.18)			
Control vs. (PCa +BPH)					
a	75 (0.67)	171 (0.76)	1.59	0.96–2.62	0.07
A	37 (0.33)	53 (0.24)			

^*^Significant at 0.05%

The frequencies for the alleles A and a of the SNP -765 G>C of the PTGS2 gene are also shown in Table 2. There is no statistically significant difference in allele frequency between the control and PCa groups (OR = 1.22, 95% CI is 0.70–2.14, *p*-value = 0.47). However, the difference between the control and BPH groups is statistically significant (OR = 2.30, 95% CI is 1.21–4.38, *p*-value = 0.01), indicating a higher prevalence of the recessive (a) allele in the BPH group (Table 2). Lastly, the difference in allele frequency between the control and the combined affected group is statistically insignificant (OR = 1.59, 95% CI is 0.96–2.62, *p*-value = 0.07) (Table 2).

### Distribution of genotypic ratios

There is no statistically significant difference in the ratios of various combinations of BB, Bb, and bb genotypes between the control and PCa groups, the control and BPH groups, and the control and combined affected groups for the SNPs -842 A>G of the PTGS1 gene (Table 3). However, there is a statistically significant difference in the ratios of certain combinations of AA, Aa, and aa genotypes between the control and the affected groups for the SNP -765 G>C of the PTGS2 gene (Table 3).

**Table 3:** Differences in the ratios of different genotype combinations for the SNP -842 A>G of the PTGS1 gene and the SNP -765 G>C of the PTGS2 gene in the control and affected groups

Genotypes	Control	Affected	OR	95% CI	*p*-value	RR	95% CI	*p*-value
SNP -842 A>G of the PTGS1 gene: Control vs. PCa								
BB/bb	53/0	59/0	1.11	0.02–57.03	0.96	1.0	1.0–1.0	N/A
BB/Bb	53/3	59/2	1.67	0.27–10.38	0.58	1.02	0.95–1.10	0.58
BB/(Bb+bb)	53/3	59/2	1.67	0.27–10.38	0.58	1.02	0.95–1.10	0.58
Bb/(BB+bb)	3/53	2/59	0.60	0.10–3.72	0.58	0.61	0.11–3.52	0.58
bb/(BB+Bb)	0/56	0/61	0.92	0.02–47.08	0.97	0.92	0.02–45.57	0.96
Control vs. BPH								
BB/bb	53/0	50/0	0.94	0.02–48.48	0.98	1.0	1.0–1.0	N/A
BB/Bb	53/3	50/1	2.83	0.28–28.12	0.37	1.03	0.96–1.12	0.35
BB/(Bb+bb)	53/3	50/1	2.83	0.28–28.12	0.37	1.03	0.96–1.12	0.35
Bb/(BB+bb)	3/53	1/50	0.35	0.04–3.51	0.37	0.37	0.04–3.40	0.38
bb/(BB+bb)	0/56	0/51	1.10	0.02–56.30	0.96	1.09	0.02–54.25	0.96
Control vs. (PCa +BPH)								
BB/bb	53/0	109/0	2.05	0.04–104.60	0.72	1.0	1.0–1.0	N/A
BB/Bb	53/3	109/3	2.06	0.40–10.54	0.39	1.03	0.96–1.10	0.43
BB/(Bb+bb)	53/3	109/3	2.06	0.40–10.54	0.39	1.03	0.96–1.10	0.43
Bb/(BB+bb)	3/53	3/109	0.49	0.09–2.49	0.39	0.5	0.10–2.39	0.39
bb/(BB+Bb)	0/56	0/112	0.5	0.01–25.64	0.73	0.5	0.01–25.10	0.73
SNP -765 G>C of the PTGS2 gene: Control vs. PCa								
AA/aa	7/26	2/28	0.27	0.05–1.39	0.12	0.31	0.07–1.40	0.13
AA/Aa	7/23	2/31	0.21	0.04–1.12	0.07	0.26	0.06–1.15	0.08
AA/(Aa+aa)	7/49	2/59	0.24	0.05–1.19	0.08	0.26	0.06–1.21	0.09
Aa/(AA+aa)	23/33	31/30	1.48	0.71–3.08	0.29	1.24	0.83–1.84	0.30
aa/(AA+Aa)	26/30	28/33	0.98	0.47–2.03	0.95	0.99	0.67–1.46	0.95
Controls vs. BPH								
AA/aa	7/26	1/34	0.11	0.01–0.94	0.04^*^	0.13	0.02–1.04	0.05
AA/Aa	7/23	1/16	0.21	0.02–1.84	0.16	0.25	0.03–1.88	0.18
AA/(Aa+aa)	7/49	1/50	0.14	0.02–1.18	0.07	0.16	0.02–1.23	0.08
Aa/(AA+aa)	23/33	16/35	0.66	0.30–1.45	0.30	0.76	0.46–1.28	0.30
aa/(AA+Aa)	26/30	34/17	2.31	1.05–5.05	0.04^*^	1.44	1.02–2.02	0.04^*^
Control vs. (PCa +BPH)								
AA/aa	7/26	3/62	0.18	0.04–0.75	0.02^*^	0.22	0.06–0.81	0.02^*^
AA/Aa	7/23	3/47	0.21	0.05–0.89	0.03^*^	0.26	0.07–0.92	0.04^*^
AA/(Aa+aa)	7/49	3/109	0.19	0.05–0.78	0.02^*^	0.21	0.06–0.80	0.02^*^
Aa/(AA+aa)	23/33	47/65	1.04	0.54–1.99	0.91	1.02	0.70–1.50	0.91
aa/(AA+aAa)	26/30	62/50	1.43	0.75–2.72	0.28	1.19	0.86–1.65	0.29

^*^Significant at 0.05 level.

For instance, the ratio of AA to aa genotypes is significantly different between the control and BPH groups (OR = 0.11, 95% CI is 0.01–0.94, *p*-value = 0.04; corresponding RR = 0.13, 95% CI is 0.02–1.04, *p*-value = 0.05). Similarly, the ratio of aa to (AA+Aa) genotypes is significantly different between the control and BPH group (OR = 2.31, 95% CI is 1.05–5.05, *p*-value = 0.04; corresponding RR = 1.44, 95% CI is1.02–2.02, *p*-value = 0.04). The difference in the ratio of AA to Aa and AA to (Aa+aa) between the control group and the PCa group is substantial but not statistically significant (OR = 0.21–0.24; *p*-values 0.08–0.09).

Lastly, the differences in some genotype ratios between the control and the combined affected (PCa + BPH) groups are statistically significant. For example, the ratio of AA to aa genotypes (RR = 0.18, 95% CI is 0.04–0.75, *p*-value = 0.02, corresponding RR = 0.22, 95% CI is 0.06–0.81, *p*-value = 0.02), and the ratio of AA to (Aa+aa) genotypes (OR = 0.19, 95% CI is 0.05–0.78, *p*-value = 0.02, corresponding RR = −0.21, 95% CI is −0.06–0.8, *p*-value = 0.02) are significantly different, suggesting a lower prevalence of the AA genotype and the A allele in the affected groups compared to the control group.

## DISCUSSION

Lebanon, a small nation with a population of 5.82 million [25], has 49.93% males, with approximately 0.65 million males aged 50 years or older [26]. Given the average male life expectancy of 77.8 years, a male tobacco use rate of 47.5%, an adult obesity rate of 32%, an alcohol consumption rate of 1.14 liters per person per year, and exposure to various pollutants, including 24.23 micrograms per cubic liter of particulate air pollutants [26], cancer poses a significant public health concern for the older male population in Lebanon.

In 2015 (the latest reporting year), Lebanon’s overall cancer incidence rate was 224.39 cases per 100,000 individuals. The top three prevalent cancers in the general population were breast cancer (43.22/100,00), lung cancer (20.71/100,00), and colorectal cancer (23.34/100,00) [27]. PCa (23.34/100,000) was the most prevalent cancer among Lebanese males [27]. There is no published report on the prevalence rate of BPH in Lebanon. The estimated prevalence of BPH in Middle Eastern countries ranges between 13.84% and 23.79% [28]. It is reasonable to assume that the prevalence rate of BPH in Lebanon falls within this range.

Given the substantial burden of prostatic disease involving cell proliferation, this study aimed to identify markers that may be common in BPH and PCa in the Lebanese male population. The focus was on examining the association of certain SNPs in the PTGS1 and PTGS2 genes with prostatic diseases, as the enzymes encoded by these genes are linked to inflammation, mitogenesis, and neoplasm [4]. Previous studies have indicated an association between specific SNPs in these genes and cancers of various organs, including the prostate gland [7–12]. The study focused on two SNPs located in the proximal regulatory region of the promoter (-842 of the PTGS1 gene and -765 of the PTGS2 gene, relative to the transcription start site) of the genes. These SNPs may overlap with binding sites for certain transcription regulators or affect local epigenetic DNA modifications, potentially influencing the overall expression of the genes.

The ratio of genotypes BB, Bb, and bb (i.e., AA, AG, and GG) for the SNP -842 A>G of the PTGS1 gene, and AA, Aa, and aa (i.e., GG, GC, and CC) for the SNP -765 G>C of the PTGS2 gene is in Hardy-Weinberg equilibrium for both the control and affected samples (Table 1). This equilibrium suggests that our sample size, although small, is adequate for genetic and epidemiologic analyses [29–31].

The frequency of the dominant (B) and recessive (b) alleles for the SNP -842 A>G of the PTGS1 gene is similar between the control and the affected populations (Table 2). However, the a (C) allele of SNP -765 G>C of the PTGS2 gene is significantly more common in the BPH group (OR = 2.30, *p*-value = 0.01), and notably more common in the combined affected group (OR = 1.59, *p*-value = 0.07) compared to the control group (Table 2). This disparity suggests a possible association of the C allele with prostatic diseases. A *p*-value of 0.07 is slightly above the conventional threshold of 0.05 for statistical significance. However, the OR of 1.59 suggests potential biological significance. Such OR and *p*-values warrant further investigation with a larger sample size or additional studies.

Further analysis of genotype ratios in the control and affected groups revealed no association between SNP -842 A>G of the PTGS1 gene and either PCa or BPH (Table 3). The b (G) allele of this SNP is very rare (1.81%) in the Lebanese population, and no homozygous recessive genotype (aa) was found in our sample. Given the small sample size, the significance of this finding is uncertain. Previous studies have also reported a lack of association between this SNP of the PTGS1 gene and other cancers, such as colorectal adenoma [32].

Our results indicate a strong association between certain genotypes of the SNP -765 G>C of the PTGS2 gene and BPH (Table 3). The ratios of AA to aa and aa to (AA + Aa) are significantly different between the control and BPH groups (*p*-value = <0.05), indicating an association between the a (C) allele and the aa (CC) genotype with BPH. In contrast, these ratios are not significantly different between the control and PCa groups (*p*-value = >0.13), although, the ratios of the genotypes AA to Aa and AA to (Aa+aa) were substantially but not significantly different (*p*-value = 0.08–0.09) between the control and PCa groups. However, the ratios of AA to aa, AA to Aa, and AA to (Aa + aa) are significantly different between the control and combined affected groups (*p*-value = 0.02–0.04), suggesting an association of the aa (CC) genotype with cell proliferative prostatic diseases such as PCa and BPH, and a protective role of the GG genotype against these diseases (Table 3). These findings underscore the importance of the PTGS2 gene’s SNP -765 G>C in the context of prostatic diseases and highlight the need for further research to understand the biological mechanisms underlying these associations.

We attempted to review the literature on the association between the SNP -765 G>C of the PTGS2 gene and the risk of BPH but found no published report. However, some studies found a link between a few other polymorphisms of the PTGS2 gene and an increased risk of BPH. For example, a previous study indicated that the polymorphism rs2745557 (distinct from SNP -765 G>C or rs20417) is linked to an increased risk of both PCa and BPH [33]. Another study found an association between four polymorphic alleles (excluding -765 G>C or rs20417) of the PTGS2 gene and an elevated risk of BPH [34]. The application of COX-2 inhibitors has been shown to alleviate BPH symptoms [35, 36], reviewed in [37], suggesting that overexpression of the COX-2 enzyme and certain PTGS2 gene alleles may be related to BPH risk.

Several studies have reported an association between the SNP -765 G>C of the PTGS2 gene and an increased risk of PCa. Some of these studies indicated that the C allele or GC genotype is linked to a higher risk of PCa [38, 39], while another study suggested that the G allele is associated with an increased risk of PCa [40]. A meta-analysis, however, found no association between SNP -765 G>C of the PTGS2 and PCa risk [41]. To resolve these conflicting findings, we examined additional reports on the association between the SNP -765 G>C of the PTGS2 gene and some other cancers. Most studies indicated the C allele, C carriers, or the GC or CC genotype to be associated with a higher risk of cancers such as pancreatic cancer [42], gastric adenocarcinoma [43, 44], and colorectal cancer [45, 46]. Several meta-analyses confirmed these findings [47–49]. However, a few studies found that the G allele, G carrier, or GG genotype is associated with certain cancers, such as gastric cancer [50] and lung cancer [51]. Additionally, some studies found no association between the SNP -765 G>C of the PTGS2 gene and the risk of certain cancers, such as breast cancer [52] and gastric cancer [53].

We reviewed the literature for studies on the effects of the two SNPs on the transcription rates of the PTGS1 and PTGS2 genes. Vogel et al. [54] reported elevated levels of mRNAs of the PTGS2 gene in subjects with the GC genotype compared to those with the GG genotype in both healthy and cancer tissues. However, the difference was not statistically significant. Notably, the study population did not include any CC genotypes. We were unable to find any reports on the effect of the SNP -842 A>G on the mRNA expression levels of the PTGS1 gene.

Overall, most (but not all) studies suggest an association between the SNP -765 G>C of the PTGS2 gene, particularly the C carrier, and an increased risk of various cancers. This apparent inconsistency may be attributed to several factors, including ethnic differences [55], the polygenic nature of phenotypic outcomes, variability in the penetrance of different contributing genes, environmental influences, epigenetic regulation, and study designs [56, 57]. Given that most complex traits and disease traits are polygenic [58] and that almost all genes are polymorphic [15], every individual or genetically homogenous population is likely to have both protective and deleterious alleles of the genes involved for any given complex trait or genetic disease [59, 60]. Therefore, association studies involving a single gene or a small number of genes may not definitively identify high-penetrance markers. A more effective approach for identifying high-penetrance genotype-phenotype association markers is genome-wide association studies (GWAS) [61, 62]. However, GWAS is technically challenging and may identify many genetic variations lacking any direct association with the phenotypes in question [63]. Thus, small-scale association studies, such as this study, on highly relevant genes to certain phenotypes remain important for validating and complementing GWAS findings.

To our knowledge, this is the first study to report a strong association between the C allele of the SNP -765 G>C of the PTGS2 gene and an increased risk of BPH among Lebanese men. Our data also suggest that the C allele of this SNP is likely associated with an increased risk of PCa. This finding supports and extends previously reported findings on the association of this polymorphic allele with an increased risk of PCa in other ethnic groups [38, 39], and various other cancers [42–49].

Although BPH is not considered a risk factor for PCa [64], both diseases share common risk factors [19, 65], and benefit from some similar therapeutic agents, such as COX-2 inhibitors [66, 67]. Based on these observations, it is plausible to suggest the prophylactic use of COX-2 inhibitors for elderly individuals with PTGS2 genotypes associated with an increased risk of proliferative prostatic diseases. However, our study has several limitations. It is an association study rather than a functional one, which means it does not explore the underlying mechanisms. In addition, the study’s small sample size of a single ethnic group presents challenges. These limitations were difficult to address due to the lack of clinical samples beyond leftover blood DNA, Lebanon’s small population, and the limited pool of older men willing to participate in genetic studies.

## MATERIALS AND METHODS

### Patients and control subjects

Patients and control subjects in this study participated in a prostate disorder screening campaign organized by Professor Asmahan El Ezzi and Wissam Zaidan in collaboration with several hospitals and medical centers in Lebanon. Each subject provided informed consent, indicating their willingness to participate in prostate-specific antigen (PSA) screening, donate a blood sample, and permit the extraction and use of DNA for genetic analysis, research, and publication. All the participants were 50 years of age or older at the time of blood sample collection. Consent was obtained following the ethical standards of the 1975 Declaration of Helsinki [68]. The present study received approval from the Institutional Review Board (IRB) of Utah Valley University (IRB approval #1013).

Participants were evaluated for prostate health by measuring serum total PSA (PSA-T) levels, followed by a digital rectal examination (DRE) as necessary. PSA-T levels were quantified using an immunoassay kit from Immunotech (Marseille, France). For subjects with PSA-T levels between 4 and 10 ng/ml (the gray zone), a free PSA test (PSA-F) was also conducted, and the PSA F/T ratio was determined to help differentiate between BPH and PCa. The International Prostate Symptom Score (IPSS) was also assessed, and trans-rectal ultrasonography was performed when appropriate. A subject was considered a control if he had normal PSA levels for two consecutive years, a normal IPSS score, and a normal DRE at the time of blood sampling. The study included 61 subjects with confirmed PCa, 51 subjects with confirmed BPH, and 56 controls with no prostatic disease.

### Molecular methods

DNA was extracted from whole blood using the QiaAmp DNA Blood Mini Kit (Qiagen, Milan, Italy). Genotyping of DNA samples was performed using the Polymerase Chain Reaction- Restriction Fragment-Length Polymorphism (PCR-RFLP) method. Briefly, a DNA fragment containing the SNP was PCR-amplified, and the amplified fragments were digested with a restriction endonuclease (RE) to detect the presence or absence of the SNP. The treated DNA samples were resolved electrophoretically, and genotypes were determined based on the resulting DNA band patterns.

For the SNP rs10306114 (i.e., -842 A to G transition) of the PTGS1 gene, the primers used were 5′-CGA TAA CTG AGC ACC TAC ATG CTG G-3′ and 5′-CCA GAC TCC ACA GCT TAC TG-3′ [32]. The 190bp amplified product was digested with the RE BaeGI (NEB, Beverly MA, USA), which recognizes the sequence 5′GKGCM/C3′ (where K is G or T, and M is A or C). For the SNP rs20417 (i.e., -765 G>C transversion) of the PTGS2 gene, the primers were 5′-CCA TCA GAA GGC AGG AAA C-3′ and (reverse) 5′-GCT CTA TAT GCA GCA CAT AC-3′ [32]. The 281bp amplified product was digested with the RE AciI (NEB), which recognizes the site 5′C/CGC3′. The authenticity of the PTGS1 and PTGS2 amplicons, as previously reported [32], was reconfirmed by their product size (190 bp and 281 bp, respectively) and restriction mapping. The PTGS1 amplicon was mapped using BaeGI, FokI, and RsaI, and the PTGS2 amplicon was mapped using AciI, MboII, and SacI (data not shown).

The PCR mixture (15 μl) contained 1× reaction buffer (containing 0.75 units of Taq DNA polymerase) (Qiagen, Germantown MD, USA), 10 picomoles of the two primers, and 10 ng of template DNA. Negative controls for PCR contained the same components except the template DNA. PCR amplification was performed using a GeneAmp 2700 thermocycler (Applied Biosystems, Carlsbad, CA, USA) with the following program: 94°C for 5 minutes (one cycle), 94°C for 45 seconds, 55–60°C for 45 seconds, 72°C for 45 seconds (35 cycles); 72°C for 5 minutes (one cycle), followed by a soak at 4°C.

To prevent cross-contamination, the PCR mixture was set in a UV-decontaminated Class IIA2 biosafety cabinet equipped with a high-efficiency particulate air (HEPA) filter using a dedicated set of pipettes. Amplified PCR products were analyzed in a space separate from the PCR setup space. Amplified DNA fragments were digested with the specified RE (0.2 unit/15 microliter reaction) overnight at 37°C, then resolved in 6% polyacrylamide gels using a vertical electrophoresis device for approximately 90 minutes at 8 volts/cm using 0.5x Tris-Borate-EDTA buffer as the electrolyte. The gels were stained for 30 minutes in ethidium bromide (0.5 μg/ml in 0.5x Tris-borate-EDTA buffer). The DNA bands were visualized on a UV transilluminator and digitally documented.

SNP genotypes were designated by the first letter of the RE used to detect the SNPs, with the genotype containing the restriction endonuclease site considered recessive. Accordingly, the genotypes for the SNP -842 A>G of the PTGS1 gene were BB, Bb, and bb (equivalent to AA, AG, and GG, respectively); and those for the SNP -765G>C of the PTGS2 gene were AA, Aa, and aa (equivalent to GG, GC, and CC, respectively).

### Statistical analyses

The frequencies of the XX, Xx and xx genotypes for an SNP in the three sample categories were analyzed in conjunction with Hardy-Weinberg (H-W) equilibrium using the χ2 goodness-of-fit test. The null hypothesis, stating that ‘the genotypes of the three populations for an SNP are in H-W equilibrium’ was rejected if the calculated χ^2^ test statistic produced a *p*-value < 0.05. The association between each SNPs polymorphic genotype and PCa and BPH was assessed by calculating the odds ratio (OR) and the 95% confidence interval (CI). In addition, the relative risk (RR) of developing PCa or BPH for each genotype, along with the 95% CI, was calculated. A *p*-value < 0.05 was considered statistically significant. The OR RR, 95% CI, and *p*-value were calculated using MedCalc Statistical Software (MedCalc, Ostend, Belgium).

## Abbreviations

BPH: benign prostate hyperplasia
CI: confidence interval
COX: Cyclooxygenases
DRE: digital rectal examination
HEPA: high-efficiency particulate air (filter)
IPSS: International Prostate Symptom Score
OR: odds ratio
PCa: prostate cancer
PCR-RFLP: polymerase chain reaction-restriction fragment length polymorphism analysis
PG: prostaglandins
PSA: prostate-specific antigen
PTGS: Prostaglandin-Endoperoxide Synthases
RR: relative risk
RE: restriction endonuclease
SNP: single nucleotide polymorphism
